# Long noncoding RNA H19: functions and mechanisms in regulating programmed cell death in cancer

**DOI:** 10.1038/s41420-024-01832-8

**Published:** 2024-02-14

**Authors:** Yuyang Xia, Tianjiao Pei, Junjie Zhao, Zilin Wang, Yu Shen, Yang Yang, Jiayu Liang

**Affiliations:** 1 Department of Urology, Institute of Urology, West China Hospital, West China School of Medicine, Sichuan University, 610041 Chengdu, China; 2 Department of Reproductive Medicine, West China Second University Hospital of Sichuan University, Chengdu, China; 3 Key Laboratory of Birth Defects and Related Diseases of Women and Children (Sichuan University), Ministry of Education, West China Second University Hospital of Sichuan University, Chengdu, China

**Keywords:** Long non-coding RNAs, Cell death, Cancer

## Abstract

Long noncoding RNAs (lncRNAs) are a group of noncoding RNAs with transcript lengths of >200 nucleotides. Mounting evidence suggests that lncRNAs are closely associated with tumorigenesis. LncRNA H19 (H19) was the first lncRNA to function as an oncogene in many malignant tumors. Apart from the established role of H19 in promoting cell growth, proliferation, invasion, migration, epithelial-mesenchymal transition (EMT), and metastasis, it has been recently discovered that H19 also inhibits programmed cell death (PCD) of cancer cells. In this review, we summarize the mechanisms by which H19 regulates PCD in cancer cells through various signaling pathways, molecular mechanisms, and epigenetic modifications. H19 regulates PCD through the Wnt/β-catenin pathway and the PI3K–Akt–mTOR pathway. It also acts as a competitive endogenous RNA (ceRNA) in PCD regulation. The interaction between H19 and RNA-binding proteins (RBP) regulates apoptosis in cancer. Moreover, epigenetic modifications, including DNA and RNA methylation and histone modifications, are also involved in H19-associated PCD regulation. In conclusion, we summarize the role of H19 signaling via PCD in cancer chemoresistance, highlighting the promising research significance of H19 as a therapeutic target. We hope that our study will contribute to a broader understanding of H19 in cancer development and treatment.

## Background

Long noncoding RNA (lncRNAs) refers to RNA molecules longer than 200 nucleotides that do not encode proteins. LncRNAs are transcribed by RNA polymerase II and are classified into enhancer lncRNAs, antisense lncRNAs, bidirectional lncRNAs, large intergenic ncRNAs, and intronic transcript lncRNAs [1, 2]. They are located in the nucleus or cytoplasm and have conserved secondary structures. LncRNAs can interact with proteins and nucleic acids and regulate gene expression at multiple levels, such as epigenetic, transcriptional, and post-transcriptional regulation, through a variety of mechanisms (e.g., gene imprinting, chromatin remodeling, cell cycle regulation, splicing regulation, messenger RNA (mRNA) degradation, and translational regulation). Continuous advancement of research has shown that lncRNAs are closely related to species evolution, embryonic development, material metabolism, and tumor occurrence, thus gaining wide attention.

Long noncoding RNA H19 (H19) was one of the first lncRNAs encoded by H19 [3]. The H19 gene is 2.5 kb in length and is located on chromosome 11p15.5, with five exons and four introns. H19 is transcribed by RNA polymerase II, spliced, polyadenylated, and exported from the nucleus to the cytoplasm [4]. The mature H19 produced by the H19 gene product is 2.3 kb in length and is called a noncoding RNA because it lacks an obvious open reading frame. H19 is abundantly expressed during embryonic development, mainly in endoderm- and mesoderm-derived tissues. However, its expression decreases after birth and is only expressed in the cardiac and skeletal muscles. Studies have shown that H19 participates in various processes, such as neurogenesis, adipocyte differentiation, lipid metabolism, angiogenesis, inflammatory reactions, cellular proliferation, fibrosis progression, and programmed cell death (PCD) [5–10]. In addition, abnormal H19 overexpression is thought to be associated with cancer development and progression, making it a potential prognostic indicator and therapeutic target for treating specific cancers. There are several different mechanisms through which H19 regulates cellular activity. For example, H19 can function as a competitive endogenous RNA (ceRNA) [11] by competitively occupying shared binding sequences of micro RNAs (miRNAs), thus sequestering miRNAs and changing the expression of their downstream target genes [11]. In addition, H19 interacts with different proteins during DNA transcription and stabilizes ribonucleoprotein complexes after RNA generation. H19 also regulates gene expression through epigenetic regulation [12–14].

PCD refers to the manner in which cells die depending on specific genes encoding signals or activities [15]. Apoptosis, autophagy, necroptosis, ferroptosis, and pyroptosis are different PCD mechanisms that are crucial for all multicellular organisms to control cell proliferation, maintain tissue homeostasis, and eliminate harmful or unnecessary cells [16]. Errors in the physiological mechanisms underlying PCD may contribute to various human diseases, particularly cancer.

Recently, H19 was found to regulate PCDs in multiple cancers, indicating a close association between H19 and PCDs [10, 12, 17–22]. Determining the potential regulatory mechanisms of H19 affecting PCD could bring about wider prospects for understanding the role of H19 in cancer development and is of great importance for cancer diagnosis and treatment. In this review, we summarize the signaling pathways, molecular mechanisms, and other regulatory mechanisms involved in H19-related PCD regulation.

## Main signaling pathways involved in H19 regulating PCD

### Wnt/β-catenin pathway

**Fig. 1:**
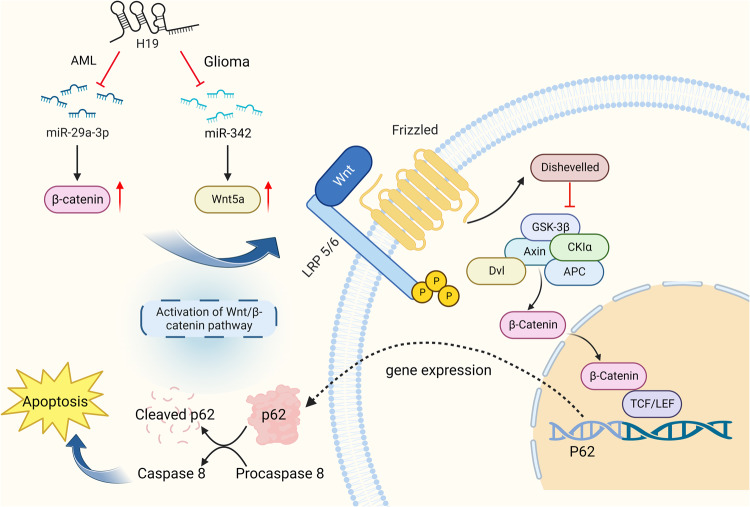
H19 regulates apoptosis through activation of the Wnt/β-catenin pathway. When Wnts are translocated to the cytoplasmic membrane, they bind to frizzled receptors (Fzd), which are members of the G-protein coupled receptors, and lead to further recruitment of scaffold proteins disheveled (Dvl) and Axin [135]. As a destruction complex of β-catenin, Axin is composed of the scaffolding protein, which also includes glycogen synthase kinase (GSK)-3b, casein kinase (CK)-1a, and adenomatous polyposis coli (APC) gene product. GSK-3b and CK1a promote Axin interactions with a co-receptor of the LRP family. Once Wnt-Fzd-Dvl-LRP5/6 structural regions are formed, LRP5/6 is phosphorylated, which initiates Wnt/β-catenin signaling and increases the expression of intranuclear β-catenin. Later on, the β-catenin binds to the T cell-specific transcription factor (TCF)/lymphocyte-enhancer-binding factor (LEF) family and regulates the gene transcription regulation through a series of complex regulatory modifications. Upregulated TCF (p62) promotes caspase 8 activation, thus activating the caspase-cascade and induces apoptosis. After H19 inhibits miR-29a-3p, the expression of intranuclear β-catenin is significantly increased and the Wnt/β-catenin pathway critical molecules T-cell factor (TCF) and lymphoid enhancer factor 1 (LEF1) were evidently up-regulated [30]. H19 also directly targets miR-342 to upregulate Wnt5a and promotes its binding with frizzled receptors [35].

Since the first member of the Wnt family was identified in 1982, studies on Wnt signaling have steadily increased [23]. Notably, the Wnt/β-catenin signaling pathway is necessary for embryonic development and adult tissue homeostasis regeneration [24]. Abnormal regulation of this pathway is closely associated with the pathogenesis of various cancers including glioma, acute myeloid leukemia, colorectal cancer, neck squamous cell cancer, non-small cell lung cancer, liver cancer etc [25–30]. The pathway is regulated by noncoding RNAs, including lncRNA H19, which directly or indirectly enhance the stability of β-catenin, thus activating the pathway [24] (see Fig. 1). By activating the Wnt/β-catenin pathway, H19 suppresses apoptosis in tumor cells [31]. Apoptosis is a form of PCD that results in the orderly and efficient removal of damaged cells while maintaining a balance among cancer cell death, survival, and genomic integrity [32]. The morphological hallmarks of apoptosis in the nucleus are chromatin condensation and nuclear fragmentation, which are accompanied by rounding of the cell, reduction in cellular volume (pyknosis), and retraction of pseudopods [33]. Membrane blebbing and ultrastructural modification of cytoplasmic organelles occur during the later stages of apoptosis along with loss of membrane integrity. Biochemically, caspases are activated, DNA and proteins break down, and phagocytes recognize altered membranes, resulting in phagocytosis [34]. The inhibition of apoptosis in cancer cells results in immortal malignant cells. The mechanism of apoptosis is complex and involves several molecular pathways. Defects at any point along these pathways lead to the malignant transformation of the affected cells, tumor metastasis, and resistance to anticancer drugs.

Accumulating evidence has shown that H19 could regulate the development of glioma by activating the Wnt/β-catenin signaling pathway [29, 35]. H19 is highly expressed in glioma and directly targets miR-342 to upregulate Wnt5a, a target gene of miR-342, to activate the Wnt5a/β-Catenin pathway and suppress tumor apoptosis [35]. Wnt5a, an important member from the Wnt family, is critical in regulating cancer cell invasion and metastasis via Wnt5a/β-catenin signaling pathway. The silence of H19 induces cell apoptosis by regulating the miR-342-mediated Wnt5a/β-Catenin signaling pathway, resulting in decreased levels of vascular endothelial growth factor A (VEGFA), MMP9, Wnt5a, and β-catenin [35].

H19 expression is markedly elevated in acute myeloid leukemia (AML). The increased expression of H19 was found to inhibit AML cell apoptosis by targeting miR-29a-3p via providing a complementary site to the 3′-UTR of miR-29a-3p [30]. Following the suppression of miR-29a-3p by H19, there is significant increase in the expression of intranuclear β-catenin, along with evident upregulation of critical molecules in the Wnt/β-catenin pathway including T-cell factor (TCF) and lymphoid enhancer factor 1 (LEF1) [30]. Upregulated TCF (p62) promotes the activation of caspase 8, the initiator caspase of extrinsic apoptosis, and an inhibitor of both necroptosis and pyroptosis [36–38].

### PI3K-Akt-mTOR pathway

**Fig. 2:**
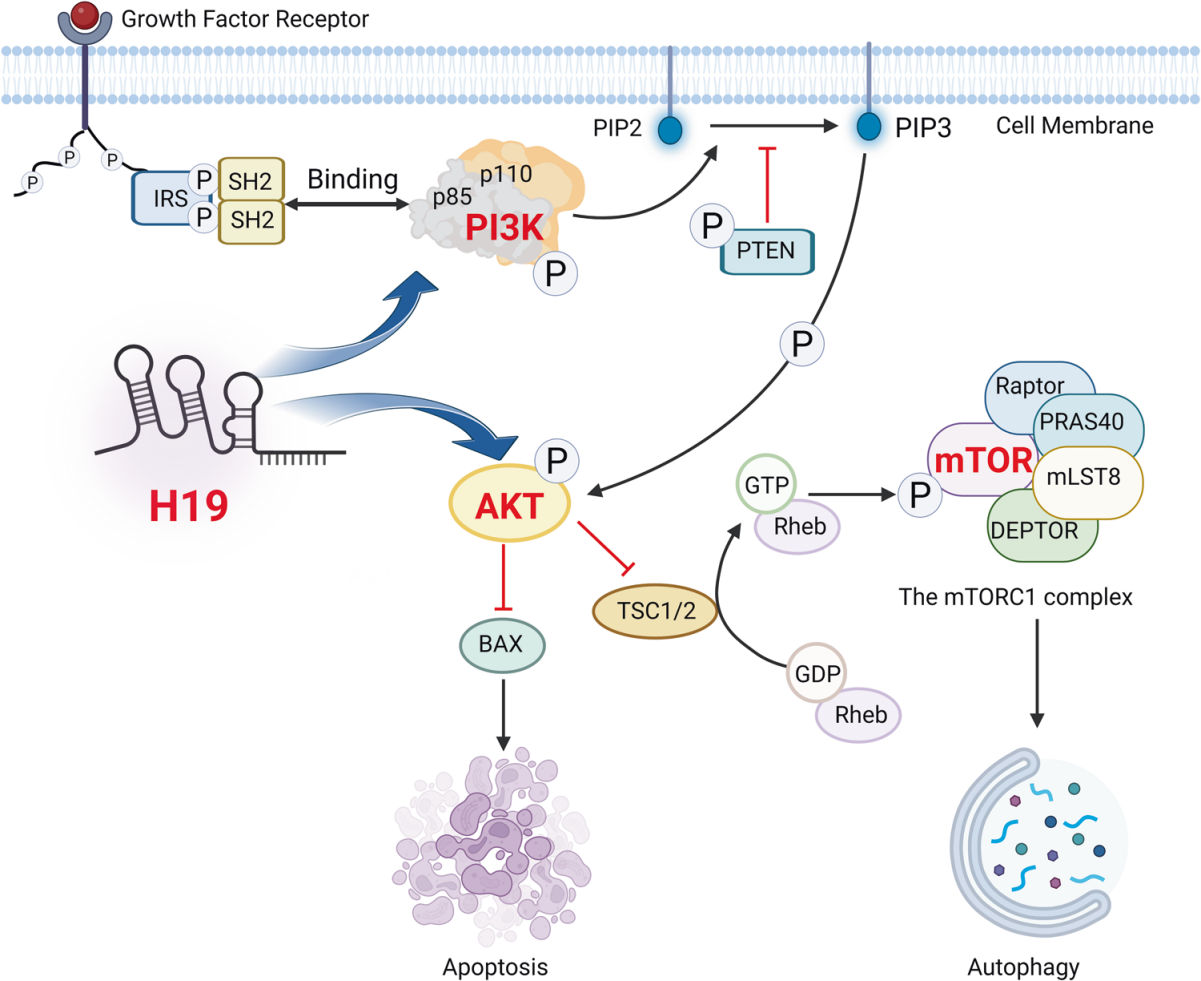
H19 regulates autophagy and apoptosis through activation of the PI3K–Akt–mTOR pathway. Phosphatidylinositol kinase (PI3K) is a dimer composed of the regulating subunit p85 and the catalytic subunit p110. When it binds with a growth factor receptor activated by growth factor, it helps the transformation from PIP2 (phosphatidylinositol 4,5-bisphosphate) to PIP3 (phosphatidylinositol 3,4,5-trisphosphate) and the phosphorylation of protein kinase B (Akt) by PIP3 [136]. The activated Akt also inhibits the activity of downstream substrates including apoptosis-related protein Bax, thereby regulating cell apoptosis. Phosphorylated Akt inhibits the transformation from Rheb bound with GDP to Rheb bound with GTP triggered by GTPase-activating protein (GAP) [48]. Failure of GTP-Rheb formation prevents activation of its target, mammalian target of rapamycin (mTOR), which forms a key regulator of autophagy mTORC1, together with Raptor, PRAS40, mLST8, and DEPTOR [137, 138]. The mTORC1 regulates different stages in the autophagy process including nucleation, autophagosome extension, autophagosome maturation and termination [139]. The lncRNA H19 takes part in PI3K–Akt–mTOR pathway via promoting the phosphorylation of PI3K and AKT, eventually inhibiting apoptosis and autophagy [45, 140]. It’s also worth noting that as a phosphatase, cancer suppressor protein PTEN can dephosphorylate Akt and reduce its activation, and can block all downstream signaling events regulated by Akt, thus is a negative regulator of PI3K [141].

The crucial roles of the phosphatidylinositol 3-kinase (PI3K)/protein kinase B (Akt)/ mammalian target of rapamycin (mTOR) signaling pathway in cell proliferation, differentiation, migration, angiogenesis, apoptosis, and other physiological processes are widely recognized [39]. Recent studies have reported a positive correlation between H19 and the PI3K/Akt pathway, which regulates apoptosis and autophagy in various cancer types. This mechanism is illustrated in detail in Fig. 2.

H19 overexpression suppresses apoptosis by activating the PI3K–Akt–mTOR pathway. In breast cancer, H19 phosphorylates Akt and increases the level of Ser473 (phosphorylated Akt). Ser473 suppresses apoptosis by downregulating the apoptosis promoter proteins Bax and cleaved caspase-3, which are key factors in apoptosis [40]. Additionally, activated Akt phosphorylates another apoptosis promoter gene, BAD, belonging to the Bcl-2 family, preventing its binding to Bcl2/Bcl-XL. This process maintains the inhibitory effect of Bcl2/Bcl-XL [41]. A similar regulatory mechanism also exists in thyroid cancer. Overexpression of H19 increases the levels of phosphorylated PI3K and Akt, thereby inducing apoptosis [42]. H19 knockout induces apoptosis in thyroid cancer cells by prominently increasing the expression of Bax and caspase-3 and suppressing the expression of Bcl-2 [40].

H19 also promotes autophagy in cancer cells via the PI3K–Akt–mTOR pathway. Autophagy is the mechanism by which cellular materials are delivered to lysosomes for degradation. Double-membrane vesicles called phagophores carry these substrates and fuse with lysosomes after developing into autophagosomes. Lysosomes then digest these substrates for recycling to create new cellular structures and/or organelles, or alternatively, for further processes and to create energy [34]. Although autophagy is often used to recycle cellular components, it can also cause cell destruction, which has been linked to the destruction of neoplastic lesions [43]. During the initial stages of tumor formation, especially when primary apoptosis is deficient, activated autophagy suppresses tumor cells. However, in the final stage of tumor development, autophagy protects tumor cells from radiation and chemotherapy [15]. The PI3K/Akt/mTOR signaling pathway has been proved to be the main regulatory pathway of autophagy [44]. The phosphorylation levels of key kinases in the PI3K/AKT/mTOR pathway are enhanced by lncRNA-H19 overexpression, which occurs in most cancer types [45]. Therefore, H19 promotes autophagy via the PI3K/AKT/mTOR pathway.

Glioma is a primary brain tumor2 thought to be derived from neuroglial stem or progenitor cells. It is the most common intracranial malignant tumor with a very poor prognosis, accounting for 30% of all primary brain tumors and nearly 80% of primary malignant tumors of the central nervous system [46]. Scientists have found that H19 is a potential tumorigenic lncRNA in glioma and have validated its contribution to the autophagy of glioma cells through the PI3K/AKT/mTOR pathway [46]. As a downstream effector of PI3K/Akt signaling, mTOR is a vital mediator of PI3K signaling that combines metabolic pathways and signal transduction in gliomas [47]. H19 overexpression inhibits mTOR phosphorylation and promotes Unc-51-like autophagy activating kinase 1 (ULK1) activation through phosphorylation. Activated ULK1 phosphorylates the Ser14 locus of Beclin-1, thereby increasing the activity of the lipase VSP34 complex and inducing autophagy. Experiments have also validated that the activation of PI3K and Akt increases mTOR activity, thereby downregulating autophagy [45]. In hepatoma cells, H19 promotes autophagy by inhibiting the PI3K–Akt–mTOR pathway [48]. The knockdown of H19 was found to decrease the levels of autophagic vesicles and the expression of the autophagy activator Beclin-1. Additionally, it increased the expression of Bcl-2 and the phosphorylation of PI3K, Akt, and mTOR, collectively inhibiting autophagy.

## H19 regulates PCD via binding with miRNAs

**Fig. 3:**
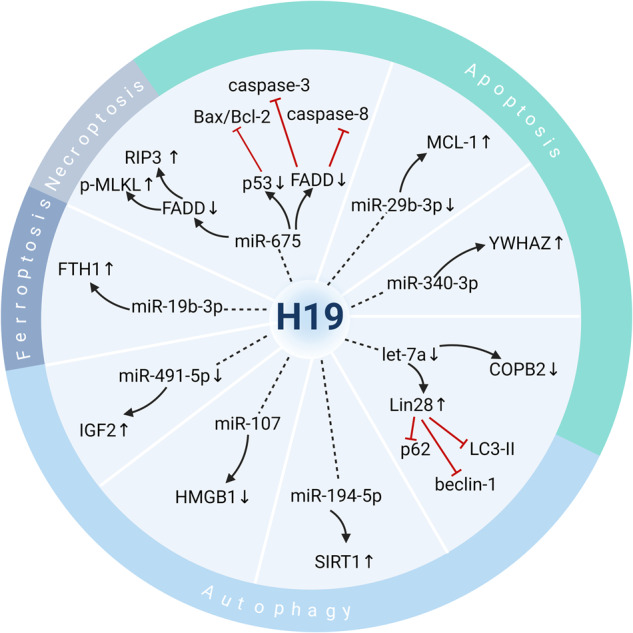
Molecular mechanisms by which H19 acts as ceRNA to regulate PCD. H19 regulates apoptosis, autophagy, ferroptosis, and necroptosis in tumor cells as a ceRNA. H19 and mRNA competitively bind with the same microRNA response elements (MRE), and H19 expression directly affects the number of miRNAs that can be bound by the corresponding mRNA. That is, H19 indirectly regulates mRNA expression through the bridge of MRE, thus regulating PCD.

In addition to the miRNA regulatory network, H19, functioning as a ceRNA, constitutes another RNA intermolecular regulatory model that involves a broader set of genes and a more extensive regulatory network for regulating PCDs in cancer. ceRNAs have recently attracted considerable attention representing a novel mode of regulation of gene expression. In 2011, Poliseno et al. proposed the ceRNA hypothesis and pointed out that ceRNAs (mRNA, lncRNA, pseudogenes, etc.) can competitively bind to the same miRNAs through microRNA response elements (MREs) to regulate gene expression levels [49]. When the same MRE exists between an lncRNA and an mRNA, they form a competitive relationship with the same type of miRNA. Given that mature miRNA is incorporated into an RNA-induced silencing complex that binds to a target mRNA, the level of intracellular lncRNA expression directly affects the number of miRNAs that can be bound by the corresponding mRNA, thus affecting miRNA-induced gene silencing (see Fig. 3). Upon upregulation of H19 in most cancer types, it competitively binds to miRNAs and prevents the formation of an RNA-induced silencing complex targeting downstream mRNAs. Abnormal expression levels of specific mRNAs contribute to the inhibition of PCDs such as apoptosis in cutaneous squamous cell carcinoma (CSCC) and autophagy in breast cancer.

In conclusion, determining the mechanisms underlying H19 as a ceRNA is helpful for identifying gene functions and regulatory mechanisms at a deeper level. It is also helpful in facilitating a deeper and more comprehensive understanding of many biological phenomena such as the regulatory network of PCD in cancer.

### H19 regulates PCD via miR-675

Overexpression of H19 in cancer cells inhibits PCD by directly targeting the target gene, miR-675. Fas-associating protein with a novel death domain (FADD) is a target of miR-675 and is involved in processes mediated by the H19/miR-675 axis. FADD expression is downregulated by miR-675. Subsequently, its downregulation inhibits the expression of its downstream proteins caspase-3 and caspase-8 [50]. p53 is another downstream protein of miR-675. Its expression is inversely associated with H19 and miR-675 but positively associated with its downstream apoptosis-related proteins Bax/Bcl-2 [51].

CSCC is the second most common nonmelanoma skin cancer [52]. The relationship between H19, miR-675, and p53 has been reported in CSCC It has been found that the overexpression of H19 upregulates the expression of its target miR-675, which subsequently downregulates the expression of the tumor suppressor gene p53 [53]. Thus, the downstream apoptosis-related proteins Bax/Bcl-2 are downregulated [54]. Considering that upregulating the expression of Bax/Bcl-2 has been proven to induce apoptosis, the overexpression of H19 in cancer cells contributes to the lack-of-p53-induced apoptosis via the H19/miR-675/p53 axis [55]. A similar pathway also exists in lung cancer, in which H19 remarkably upregulates miR-675 and thus suppresses p53 and Bax, collectively inhibiting apoptosis [51].

Gliomas are highly malignant tumors that are prone to metastasis and have a poor prognosis. H19 expression in glioma tissues is higher than that in para-carcinoma tissues and is associated with poor prognosis in glioma patients [29]. Therefore, the miRNA derivative of H19, miR-675, is also upregulated. The vitamin D receptor (VDR) is a target gene of miR-675 and is suppressed by miR-675, thus downregulating the expression of VDR by the upregulation of miR-675 [56]. Furthermore, H19 reduces the mRNA and protein levels of VDR [56]. Notably, VDR has been found to protect against glioma by inducing apoptosis [56, 57]. Thus, H19 inhibits glioma cell apoptosis through the H19/miR-675/VDR feedback loop.

The molecular pathways involved in H19-induced apoptosis in hepatoblastoma cells were discovered, and it was found that H19 suppressed the growth of hepatoblastoma cells by promoting apoptosis via the miR-675/FADD and miR-138/PTK2 signaling pathways [58]. Overexpression of H19 downregulates the expression of FADD by targeting miR-675 and upregulates the expression of PTK2 by targeting miR-138. Finally, both the increased expression of PTK2 and reduced expression of FADD lead to the inhibition of cell apoptosis, thus promoting the tumorigenesis of hepatoblastoma.

Hepatocellular carcinoma (HCC) is the most common cause of cancer-related deaths and is a malignancy of the liver. LncRNAs have been identified as effective modulators of carcinogenesis and their abnormal expression is related to the initiation, progression, and metastasis of HCC cells [59, 60]. Knockdown of H19 increases apoptosis in HCC cells. The results of P53 protein analysis showed that knockdown of H19 and its derivative miR-675 induced the expression of p53, eventually promoting apoptosis in MHCC97H cells [61]. Because H19 is dramatically upregulated in HCC cells, p53 is suppressed via the H19/miR-675 axis, thereby inhibiting tumor cell apoptosis. In addition, H19 inhibits the apoptosis of HCC cells treated with propofol by upregulating LIMK1 via sponging miR-520a-3p [62]. Its knockdown inhibits cell proliferation and promotes apoptosis by upregulating miR-15b in HCC cells [59].

Although H19 is upregulated in most tumor cells, H19 is downregulated in isolated cancer types such as nephroblastoma. Lower gene expression levels of lncRNAs H19 and miR-675 (*p* < 0.05) were observed in nephroblastoma cells, followed by higher gene expression levels of TGFBI than that in normal cells [63]. H19 inhibits growth and induces morphological changes in nephroblastoma cells. Downregulation of H19 suppresses TGFBI expression by regulating miR-675 levels, thereby promoting apoptosis in nephroblastoma cells.

In addition to inhibiting apoptosis in cancer cells, H19 triggers necroptosis by influencing miR-675 expression [22]. Necroptosis is a new form of programmed necrosis mediated by the necrosome, which is a complex formed by the kinase receptor-interacting proteins RIP1 and RIP3 [64, 65]. RIP1 phosphorylates RIP3, which in turn phosphorylates Mixed Lineage Kinase Domain-Like Pseudokinase (MLKL), which is recruited to the necrosome [66]. Phosphorylation leads to MLKL oligomerization, which disrupts membrane integrity and subsequently results in cell death [67]. In liver cancer, miR-675 is upregulated along with the overexpression of H19, which suppresses FADD. Activated caspase 8, which depends on FADD, cleaves RIP1 and RIP3 kinases and is thus responsible for inhibiting necroptosis [68]. Accordingly, cells deficient in FADD facilitate necroptosis in liver cancer, as they are unable to recruit and activate procaspase 8. Moreover, miR-675 increases the levels of both Mixed Lineage Kinase Domain-Like Pseudokinase (p-MLKL) and RIP3, which are key signaling molecules in necroptosis, thus promoting liver necroptosis in response to inflammatory signals [22].

### H19 regulates PCD via let-7

Coatomer protein complex subunit beta 2 (COPB2) is a protein that functions to transport other proteins as vesicles from the endoplasmic reticulum to the Golgi apparatus [69]. Previous studies have indicated that a reduction in COPB2 expression inhibits cell growth and induces apoptosis through the JNK/c-Jun signaling pathway in RKO and HCT116 cells [70]. In gastric cancer, COPB2 is regulated by let-7a, which acts as a molecular sponge for H19 [71]. According to the experimental results, let-7a expression was reduced in the H19 overexpression group, while let-7a overexpression was found in the H19 knockdown group [71]. Moreover, it has been proven that reduced expression of COPB2 induces cellular apoptosis and inhibits cell growth and invasion in gastric cancer, similar to previously studied tumors [71]. In conclusion, H19 regulates the expression of the apoptosis inducer, COPB2, by sponging let-7a.

Breast cancer (BC) is one of the leading causes of cancer-associated mortality in females aged ≤40 globally [72]. H19 is significantly upregulated in BC cells, and plays an oncogenic role in the progression of BC metastasis. The overexpression of H19 decreases the expression of autophagy-associated molecules (beclin-1 and LC3-II) in MDA-231 cells. Moreover, the H19/let-7/Lin28 loop is required for the downregulation of autophagy in BC cells [73]. Overexpressed H19 may act as a sponge to antagonize miRNA let-7 [74]. Due to the existence of a double-negative feedback loop between let-7 and its RNA-binding protein Lin28 [75], the suppression of let-7 by H19 contributes to the upregulation of Lin28. The inhibitory effect of Lin28 on the autophagy-associated molecules p62, beclin‑1 and LC3-II was observed in a previous study [73]. Collectively, lncRNA H19, miRNA let-7, and transcription factor Lin28 may form a double-negative ceRNA network in BC, inhibiting autophagy in BC cells by regulating downstream autophagy-associated molecules.

### H19 regulates PCD via miR-340-3p

In addition to let-7, H19 suppresses apoptosis in BC cells by interacting with miR-340-3p [76]. Mechanistically, H19 competitively binds to miR-340-3p, which enhances apoptosis. As a direct target of miR-340-3p, tyrosine 3-monooxygenase/tryptophan 5-monooxygenase activation protein zeta (YWHAZ) is upregulated in BC tissues. YWHAZ suppresses apoptosis by encoding anti-apoptotic proteins from a highly conserved dimeric protein family. In conclusion, overexpression of H19 in BC tissues sponges miR-340-3p, thus upregulating its downstream anti-apoptotic protein YWHAZ and consequently suppressing apoptosis.

### H19 regulates PCD via miR-29b-3p

Multiple myeloma (MM) is a malignant tumor characterized by the accumulation of large quantities of malignant plasma cells in the bone marrow and the presence of monoclonal proteins (M proteins) in the blood, urine, or both [77]. The upregulated lncRNA H19 in MM acts as a miRNA sponge to suppress miR-29b-3p, thereby enhancing the transcriptional translation of MCL-1, a downstream protein of miR-29b-3p [78]. Mcl-1 is an anti-apoptotic protein that, similar to Bcl-2 and Bcl-XL, negatively regulates intrinsic apoptotic pathways [79, 80]. Therefore, upregulated H19 inhibits apoptosis in MM cells via the H19/miR-29b-3p/MCL-1 axis. Notably, the anti-apoptotic effect was found to play an important role in drug resistance, providing novel insights into drug resistance via the H19/miR-29b-3p/MCL-1 axis [78].

### H19 regulates PCD via miR-194-5p

Colorectal cancer (CRC) is the third leading cause of cancer-related deaths worldwide and is marked by poor prognostic outcomes and complexities in treatment [81]. Accumulating evidence has shown that H19 is upregulated in CRC cells and is significantly associated with poor recurrence-free survival. H19 also promotes tumor growth by recruiting and binding to eIF4A3 [82]. In CRC, H19 functions as a ceRNA of miR-194–5p, a suppressive miRNA of SIRT1. H19 harbors a recognition sequence for miR-194-5p, which enables lncRNAs to competitively bind to miR-194-5p. As SIRT1 is a potential target gene of miR-194-5p, the miR-194-5p-mediated repressive activity of SIRT1 is abolished by H19, consequently triggering autophagy in CRC tissues [81]. In conclusion, H19 induces autophagy in CRC cells by upregulating SIRT1 expression through sponging miR-194-5p.

### H19 regulates PCD via miR-107

Head and neck cancer ranks sixth among the most common tumors worldwide, with laryngeal cancer accounting for the largest proportion. HMGB1 is a target of miR-107 in laryngeal squamous cell carcinoma (LSCC) cells and its knockdown suppresses autophagy in LSCC cells. In addition, miR-107 acted as a target of H19, and the inhibitory effects of H19 shRNA on autophagy were reversed by the miR-107 inhibitor. Interference of H19 by short hairpin RNA (shRNA) effectively suppresses high autophagy levels in LSCC cell lines [83]. In conclusion, H19 suppresses autophagy in LSCC cells through the H19/miR-107/HMGB1 axis.

### H19 regulates PCD via miR-491-5p

Glioblastoma multiforme (GBM) is the most common brain tumor and has a high rate of therapeutic resistance and recurrence. Autophagy plays a vital role in GBM, allowing tumor cells to thrive under hypoxia and toxic stress [84]. H19 is highly expressed in GBM tissues, is associated with poor prognosis, and promotes GBM progression and angiogenesis [85, 86]. H19 regulates autophagy in GBM by sponging miR-491-5p and binding to specific sites, including the seed region of miR-491-5p, to exert antagonistic effects on miR-491-5p [84]. According to previous studies, miR-491-5p inhibits the autophagy signaling pathway by targeting insulin-like growth factor 2 (IGF2) [87] and participates in autophagy in GBM by regulating the MAPK, PI3K/Akt, and mTOR pathways [88, 89]. In conclusion, upregulation of H19 promotes autophagy by sponging miR-491-5p.

### H19 regulates PCD via miR-19b-3p

Similar to many other cancer types, lncRNA H19 is upregulated in lung cancer cells and functions as a ceRNA that binds with miR-19b-3p to sponge RNA [90]. This mechanism enhances the transcriptional activity of ferritin heavy chain 1 (FTH1), an endogenous target of miR-19b-3p. FTH1 is an iron metabolism-related gene and a critical mediator of ferroptosis.

Ferroptosis is an iron-dependent form of non-apoptotic cell death triggered by the accumulation of cytosolic and lipid iron-dependent reactive oxygen species (ROS) [91]. It plays a pivotal role in the suppression of tumorigenesis by removing cells that are deficient in key nutrients in the environment or are damaged by infection or ambient stress. Numerous studies have confirmed the pivotal role of ferroptosis in killing cancer cells and suppressing cancer growth [91]. Thus, H19 inhibits ferroptosis in lung cancer cells via the H19/miR-19b-3p/FTH1 axis, thereby regulating cancer development.

## H19 regulates apoptosis via interacting with RNA binding protein (RBP)

Noncoding RNA sequences, including lncRNAs, perform various functions by directly interacting with RNA-binding proteins (RBP). RBPs bind to specific RNAs and regulate their expression at the RNA level. It has been shown that lncRNAs combine with RBPs to influence their functions [92]. Conversely, specific RBPs can interact with lncRNAs to influence their function or regulate their expression at the transcriptional level to regulate downstream gene expression [93]. The interaction between RNAs and proteins is the key to cellular homeostasis, and perturbations in RNA-RBP interactions can lead to cellular dysfunction or even cell death [94, 95]. Several studies have shown that lncRNA-RBP interactions are related to the regulation of apoptosis [96–99].

It has been shown that m^5^C-modified H19 lncRNA can be specifically bound by G3BP1 (Ras-GTPase-activating protein binding protein 1), a well-known oncoprotein.

This leads to MYC accumulation and MYC-driven apoptosis [100, 101]. In HCC, methylation of H19 is abnormally elevated and affects its stability, disturbing the interaction of lncRNA H19 with the protein G3BP1 [100]. Aberrant interactions between RNA and proteins in HCC affect MYC accumulation and induce apoptosis.

## H19 regulates PCD through DNA methylation

DNA methylation was the first discovered epigenetic modification [102–104], which consists of the covalent addition of a methyl group to the 5-carbon of cytosine, forming 5-methylcytosine (5mC) [105]. In humans, DNA methylation occurs almost exclusively within CpG dinucleotides [106], approximately 60–80% of which are methylated in the human genome, except for some regions rich in CpG islands. DNA methylation acts as a repressive epigenetic marker. However, the functions of DNA methylation vary depending on the genomic context [106, 107]. DNA methylation in proximal and distal regulatory elements suppresses transcription by altering the binding of transcription factors and/or recruiting enzymes to modify the chromatin structure. Conversely, DNA methylation of gene bodies may enhance transcriptional elongation and affect splicing. In case of densely methylated repetitive elements, DNA methylation is the major repression mechanism.

### H19 regulates autophagy through DNA methylation

**Fig. 4:**
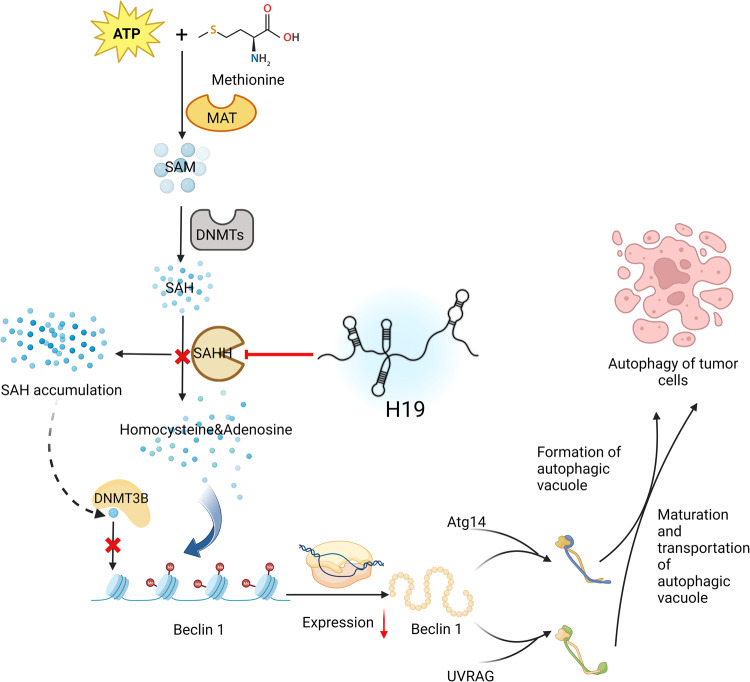
DNA methylation mechanisms of H19 regulating autophagy. Normally, S-adenosylhomocysteine (SAM) is biosynthesized by methionine and MAT (methionine adenosyl transferase) utilizing ATP for energy supply. Then SAM is converted to SAH (S-adenosylhomocysteine) catalyzed by DNMTs. SAH will be cleaved by S-adenosylhomocysteine hydrolase (SAHH) into homocysteine and adenosine. However, when overexpressed H19 in cancer cells binds with SAHH and inhibits its function, the cleavage of SAH is inhibited, leading to its accumulation [13]. The surplus SAH binds with DNMT3B to prevent its binding with Beclin1, thus allowing its expression. Otherwise, Beclin1 will lose its transcriptional activity by DNMT3B-mediated methylation. The Beclin1 protein then binds with Atg14 or UVRAG to promote autophagy via regulating the formation of autophagic vacuole and maturation and transportation of autophagic vacuole in tumor cells [142, 143].

Methylation plays a role in the regulation of many tumors. In BC, H19 regulates autophagy by regulating the related gene Beclin1 via epigenetic regulation [12] (see Fig. 4). The autophagy-related gene Beclin1 is positively correlated with H19, and is upregulated in BC cells along with the upregulation of H19. Previous studies demonstrated that H19 binds and inhibits S-adenosyl homocysteine hydrolase (SAHH), which consequently decreases DNMT3B-mediated methylation [13]. The mechanism by which H19 regulates Beclin-1 has been identified. DNMT3B directly binds to regions of the Beclin1 promoter, thus increasing the methylation of these regions, and H19 knockdown promotes this interaction [12].

### H19 regulates apoptosis through DNA methylation

In lung adenocarcinoma, H19 is responsible for tumor progression by mediating methylation-dependent repression of CDH1 promoter [14]. Both H19 and CDH1 methylation were upregulated in lung adenocarcinoma tissues. Silencing H19 inhibits the methylation of CDH1, which suppresses cell proliferation, sphere-forming ability, apoptosis, migration, and invasion [14].

## Other molecular mechanisms of H19

In addition to the mechanisms mentioned above, H19 can also act as an important epigenetic regulator in tumors. Accumulating evidence has clarified the mechanisms underlying the epigenetic modifications associated with H19. This process involves H19, which mediates the methylation and histone modification of its downstream molecules, and the epigenetic modification of H19 by other molecules.

### Methylation of H19 regulates PCDs in cancer

Since the discovery of N6-methyladenosine (m6A) in mRNA, 160–170 types of RNA modifications have been identified, including m6A, 5-methylcytosine (m5C), and 7-methylguanylate [108]. As a reversible epigenetic modification, m5C modification of RNA influences the modified RNA molecules and plays important roles in various biological processes, including RNA stability control, protein synthesis, and transcriptional regulation. NSUN2 (NOP2/Sun domain family, member 2), a methyltransferase responsible for the m5C modification of RNAs, has been reported to be highly expressed in multiple tumors [100]. M5C modification of lncRNA H19 mediated by NSUN2 increases the stability of H19 and elevates its expression [109]. Abnormally elevated H19 displays carcinogenic effects by regulating PCDs in cancer cells.

### Histone modification of H19 regulates PCDs in cancer

Histone modification is another regulatory mechanism in addition to DNA and RNA methylation. Histone proteins are subjected to a large variety of post-translational modifications, which, alone or in combination, characterize and shape functional chromatin states [110].

In CRC, histone deacetylases (HDACs) catalyze the removal of acetyl groups from lysine residues in histones to suppress gene transcription. HDAC2 is frequently downregulated in metastatic CRC tissues. It attenuates H19 transcriptional activation, and the underlying mechanism has been elucidated. HDAC2 binds to the H19 promoter and its deletion promotes the acetylation of histone H3K27 in DLD1 HDAC2 KO cells. Additionally, ChIP experiments have confirmed that the level of acetylated histone H3K27 at the H19 promoter is increased in DLD1 HDAC2 KO cells [111]. HDAC2 inhibits H19 expression via histone H3K27 deacetylation in its promoter by binding to SP1 [111]. As previously described, H19 sponges miR-194-5p as a ceRNA, through which H19 modulates SIRT1 expression, thus inducing autophagy in CRC. In conclusion, HDAC2 inhibits H19 via histone deacetylation, and HDAC2 downregulation in CRC cells leads to the upregulation of H19, consequently inducing autophagy.

## H19-associated PCD-induced treatment resistance

Resistance to treatment is the leading cause of cancer-related deaths worldwide and remains a major obstacle in clinical management. It occurs when cancer cells fail to respond to conventional therapeutics, such as chemotherapy, immunotherapy, and radiotherapy, through multiple molecular mechanisms [112–114]. While the overexpression of “onco-suppressor” lncRNAs endorses chemosensitivity, “oncogenic” lncRNAs promote chemoresistance by acting as mediators of MDR [115–117]. This process involves various mechanisms, including alteration of drug concentration [118], regulation of apoptotic proteins [119], and activation of autophagy [120]. A previous study found that lncRNA H19 was abnormally expressed in most treatment-resistant processes [121]. It may inhibit tumor cell apoptosis and hinder the apoptotic function of antineoplastic drugs by controlling relevant signal transduction pathways, such as the Wnt/β-catenin and PI3K/AKT cascades, and by interacting with relevant miRNAs [122]. This section describes the current research progress on H19-associated molecules that regulate PCD to overcome cancer resistance and provides novel insights into the clinical development of H19-targeted treatment for cancer resistance.

### H19 and autophagy-mediated cancer resistance

In LSCC, the H19/miR-107/HMGB1 axis sensitizes cancer cells to platinum-based drugs such as cisplatin (CDDP) by suppressing autophagy in vitro and in vivo [83]. As a target of H19, miR-107 is suppressed in LSCC, upregulating its downstream molecule HMGB1. Researchers have found that the knockdown of HMGB1 inhibits autophagy by inhibiting the level of Beclin1 and suppressing the conversion of LC3B-I to LC3B-II. This enhances cisplatin sensitivity, while H19 knockdown inhibits autophagy-mediated drug resistance through the aforementioned H19/miR-107/HMGB1 axis.

In BC, H19 is a critical inducer of TAM resistance, which promotes autophagy by regulating the AHH/DNMT3B axis [12]. H19 is upregulated in tamoxifen-resistant BC cell lines and promotes resistance to tamoxifen by inducing autophagy, thereby facilitating tamoxifen resistance. H19 regulates the autophagy-related gene Beclin-1 via the H19-SAHH-DNMT3B axis through epigenetic regulation, which further affects tamoxifen resistance in MCF7/TAMR cells [12].

miR-615-3p is a target of H19 and binds to ATG7 in non-small cell lung cancer (NSCLC). Exosomal H19 affected erlotinib resistance in erlotinib-resistant NSCLC cells by targeting miR-615-3p to regulate ATG7 expression. In addition, serum exosomal H19 levels were upregulated in patients with erlotinib resistance. Furthermore, the downregulation of H19 decreases the resistance of tumor cells to erlotinib in vivo [123].

### H19 and apoptosis-mediated cancer resistance

H19 is involved in CRC resistance to MTX-mediated antimetabolite action via modulation of miR-186/CPEB2, miR-760/PPP1R1B [124, 125], and WNT/β-catenin [126], thus regulating apoptosis via the mechanism mentioned above.

## H19 as a therapeutic target

Given its abnormal expression levels and diverse functions in several human cancers, H19 has aroused extensive interest regarding its implications in disease pathophysiology and its potential application as a therapeutic target in cancer. RNA interference (RNAi) technology based on shRNAs, small interfering RNAs (siRNAs), antisense oligonucleotides (ASOs), and clustered regulatory interspaced short palindromic repeats/CRISPR-associated protein 9 (CRISPR‒Cas9) are accessible genetic tools that can be used to target H19 to inhibit cancer progression and sensitize the therapeutic response to chemotherapy, thus improving clinical outcomes [127].

According to previous studies, H19 inhibition using shRNA [128], siRNA [61], CRISPR/Cas9 system [61], and site-specific ASOs [129] may suppress tumor growth, metastasis, and invasiveness. Therefore, inhibition of H19 expression using RNAi technology and the CRISPR‒Cas9 system is a promising strategy for cancer treatment.

In addition, a DNA plasmid named H19-DTA (also known as BC-819), which targets the expression of a fragment of the diphtheria toxin (DT-A) under the control of the H19 promoter, provides a feasible novel therapeutic option for H19-targeted cancer therapy [130, 131]. Furthermore, H19-DTA-P4-DTA, a double promoter vector, possesses stronger anticancer activity than single promoter vectors [127].

Two early clinical trials have been conducted to explore the efficacy and safety of H19-DTA in patients with recurrent ovarian/peritoneal cancer or invasive bladder cancer [132, 133]. Researchers have found that H19-DTA exhibits a good safety profile, inhibits new tumor growth, stabilizes progression, and prolongs the time to recurrence, making it a promising medication for cancer treatment.

In conclusion, as H19-targeted therapies are viable and show satisfactory antitumor outcomes, research on H19 has great clinical value.

## Conclusion

Since its discovery, numerous studies have focused on the role of H19 in the pathogenesis of various types of cancer through different mechanisms, such as sponging miRNAs, interactions with proteins, and epigenetic modifications. In this review, we focused on the role of H19 in cancer through the modulation of PCD. We summarized the molecular mechanisms by which H19 regulates apoptosis and autophagy in the pathogenesis of cancers via its regulatory function in several oncogenic signaling pathways, such as the PI3K/Akt and canonical Wnt/β-catenin pathways, and through the H19/miR-675 axis (see Tables 1 and 2). By directly acting on key molecules or indirectly altering the levels of downstream proteins associated with these signaling pathways, highly expressed H19 regulates PCD, thereby influencing cancer development. Based on these results, H19 may be a promising therapeutic target or biomarker for the diagnosis, prevention, treatment, and prognosis of cancer. Although the association of H19 with various oncogenic signaling pathways in the regulation of PCD in cancer has been discussed in this review, certain deficiencies and problems remain, and further studies are needed to strengthen this link. As mentioned above, one of the mechanisms by which H19 regulates PCD is through epigenetic modifications [134]. However, few studies have focused on the association of H19 with epigenetic regulation. Therefore, further studies are warranted in this regard. Moreover, although H19 is highly expressed in most cancers and has carcinogenic functions, it may play a dual role in cancer. Reports suggest the downregulation of H19 in cancer tissues and its function as a suppressor gene also exist. The reason for the discrepant results observed in the H19 expression needs to be further investigated.

**Table 1:** Molecular mechanisms by which H19 regulates apoptosis in multiple tumors.

PCD type	Tumor types	H19 expression	Signaling pathway	Molecular Mechanisms	Cell processes	References
Apoptosis	Acute myeloid leukemia (AML)	Up regulation	Wnt/β-catenin	↑ID2; ↓miR-29a-3p, ↑β-catenin, ↑T-cell factor (TCF), ↑lymphoid enhancer factor 1 (LEF1)	Promote proliferation, migration and invasion; Inhibit apoptosis	[43, 44]
Apoptosis	Breast cancer (BC)	Up regulation	Akt; Wnt/β-catenin	↑Bax, ↑cleaved caspase-3; ↓p-Akt (Ser473), ↓Bcl-2 ↓BIK; ↓MiR-138, ↑SOX4	Promote proliferation, progression and EMT; Facilitate drug resistance; Suppress apoptosis	[33, 34, 52, 68]
			H19/miR-340-3p	competitively binding miR-340-3p, regulating tyrosine 3-monooxygenase/tryptophan 5-monooxygenase acti-vation protein zeta (YWHAZ) and potentiate the Wnt/β-catenin signaling		
Apoptosis	Cutaneous squamous cell carcinoma (CSCC)	Up regulation	H19/microRNA‑675 axis	↑miR-675, ↓p53	Promote EMT, proliferation, invasion	[69]
Apoptosis	Esophageal cancer (EC)	Up regulation	let-7c/STAT3/EZH2/β-catenin axis.	↑STAT3, ↑EZH2, ↑β-catenin	Promote EMT, metastasis, migration, and invasion	[70]
Apoptosis	Gastric cancer (GC)	Up regulation	miR-138/E2F2 axis	↓p53; ↓let-7a; ↓miR-138, ↑E2F2	Increase proliferation; Inhibit apoptosis	[37, 71, 72]
Apoptosis	Glioma	Up regulation	Wnt/β-catenin; Negative feedback loop of H19/miR‐675/VDR	↑CREB1; ↑DVL2,↑cyclinD1, ↑β-catenin； ↓miR-152； ↓protein level of vitamin D receptor (VDR); ↓miR-140, ↑inhibitor of apoptosis-stimulating protein of p53 (iASPP)	Prompt invasion, proliferation, radio-resistance; Inhibit apoptosis	[57, 73, 74]
Apoptosis	Hepatoblastoma	Up regulation	miR-675/FADD, miR‐138/PTK2;	↑:H19, miR‐675, PTK2, HIFIA, FAK (HBV +), ↓: miR-138, FADD, caspase‐8, caspase‐3(HBV+);	Promote metastasis, progression	[59]
Apoptosis	Hepatocellular carcinoma (HCC)	Up regulation	MAPK/ERK; miR-520a-3p/LIMK1 axis; miR-15b/CDC42/PAK1 axis; EMT	↑Protein levels of MAPK and ERK; ↓p53; ↓miR-520a-3p, ↑LIMK1; ↑CDC42, ↓ miR-15b; ↓ EMT pathway-related genes (N-cad-herin, Vimentin, β-catenin, MMP-9)	Suppress oxidative stress (OS); Reverse chemotherapy resistance of CD133þ cancer stem cells; Inhibit apoptosis; Promote EMT, proliferation, migration, invasion	[62, 63, 75, 76]
Apoptosis	Lung cancer	Up regulation	H19/miR-29b-3p/STAT3	↓miR-29b-3p; ↓EMT-specific proteins: vimentin, Snail, Slug (↑epithelial cadherin); ↑miR‐675‐5p, ↓p53, ↓Bax, ↑ Bcl‐2	Promote proliferation, EMT; Decrease the survival rate and viability	[55, 65]
Apoptosis	Multiple myeloma (MM)	Up regulation	H19/miR-29b-3p/MCL-1	↓ miR-29b-3p, ↑MCL-1 Transcriptional translation	Reduce cell sensitivity to the chemotherapeutic drug BTZ; Inhibit apoptosis	[39]
Apoptosis	Nephroblastoma	Down regulation	miR-675/TGFBI axis	↓H19, miR-675; ↑TGFBI	Inhibits proliferation; Enhance apoptosis; Induce growth inhibition and morphological changes	[66]
Apoptosis	Ovarian cancer (OC)	Up regulation	H19 → EZH2 → p21/PTEN; PI3K/AKT/mTOR	↑p21 and PTEN protein levels; ↓ EZH2 protein; ↓serum miR-140- 5p, ↓ p-PTEN, ↑ p-AKT (Thr308), p-AKT (Ser473), p-mTOR (Ser2448), p-FoxO1 (Ser256), p-MDM2 (Ser166), p-NF-κB p65 (Ser536), and p-GSK-3β (Ser9)	Suppress apoptosis, cell mortality, drug sensitivity; Promote invasion, Migration, EMT	[77, 78]

**Table 2:** Molecular mechanisms by which H19 regulates PCDs other than apoptosis in multiple tumors.

PCD type	Tumor types	H19 expression	Role	Signaling pathway	Molecule Mechanism	Cell processes	References
Autophagy	Adrenocortical carcinoma (ACC)	Down regulation	A prognostic factor for the poor overall survival of patients	-	↓ H19, counterbalanced by ↑IGF2, leads to ↓autophagy markers, high proliferation rate and metastatic potential in ACC patients	Inhibit tumor progression	[21, 27]
Autophagy	Bladder cancer (BC)	Up regulation	Oncogene; Suppress the interaction between NEDD4L and ULK1	-	↑ULK1	Aggravate autophagy	[28]
Autophagy	Breast cancer	Up regulation	Oncogene; Epigenetic regulation	H19/let-7/Lin28 ceRNA network	↓let-7, ↑H19, ↑Lin28	Down-regulate autophagy; Promote EMT, migration and invasion; Promote resistance	[5, 23]
Autophagy	Colorectal cancer (CRC)	Up regulation	Mediate the activation of Wnt/β-catenin pathway in MTX resistance of CRC cells; Sponge miR-194–5p as ceRNA to modulate SIRT1 expression	Wnt/β-catenin	↑β-catenin; ↑SIRT1	Improve the MTX resistance; Promote autophagy; Confer 5-Fu resistance	[29, 30]
Autophagy	Glioblastoma (GBM)	Up regulation	Oncogene	Sponging miR-491-5p	↓miR-491-5p	Activate autophagy; Promote proliferation, progression	[25]
Autophagy	Glioma	Up regulation	Oncogene	mTOR/ULK1	↑the ratio of p-mTOR/mTOR; ↓the ratio of p-ULK1/ULK1; ↑the ratio of LC3-II/I, ↑Beclin-1 protein levels	Promote occurrence, development, proliferation, migration, autophagy	[31]
Autophagy	Hepatoma carcinoma	Up regulation	Down-regulating PI3K–Akt–mTOR pathway	PI3K–Akt–mTOR	↓Beclin-1, ↓ the ration of LC3-II/LC3-I, ↑ p62; ↑the phosphorylated activity of PI3K, Akt and mTOR	Up-regulate autophagy, suppress cell viability	[20]
Autophagy	Laryngeal squamous cell carcinoma (LSCC)	Up regulation	Suppresses autophagy level and drug resistance	H19/miR-107 / HMGB1 axis	↓miR-107, ↑HMGB1,	H19 shRNA suppresses autophagy and cisplatin resistance	[26]
Necroptosis	Acute myeloid leukemia (AML)	Up regulation	Activate the Wnt/β-catenin pathway	H19/miR-29a-3p/β-catenin	↓miR-29a-3p, ↑β-catenin, ↑T-cell factor (TCF), ↑caspase 8	Inhibit apoptosis, necroptosis, pyroptosis	[21–23]
Necroptosis	Liver cancer	Up regulation	Suppress Fas-associated protein with death domain (FADD)	H19/miR-675/FADD	↑miR-675, ↓FADD	Induce necroptosis, suppress cancer growth	[51]
Ferroptosis	Lung cancer	Up regulation	Competitively bind with miR-19b-3p	H19/miR-19b-3p/FTH1	↓miR-19b-3p, ↑Ferritin heavy chain 1 (FTH1)	Suppress ferroptosis	[75]
